# Obituary Notices

**Published:** 1838

**Authors:** 


### Art. VII.—Obituary Notices.

It becomes our duty to announce the recent death of several distinguished members of the profession, both foreign and American.

*Dr. McIntosh.*—This eminent Scotch physician, lecturer and writer, died in October last, at his residence in Edinburgh. We copy from the London Medical Gazette, for November, the following notice of this event:

"We regret to have to announce the decease of Dr. Mackintosh, of Edinburgh, which took place last week, in consequence of an attack of typhus fever. Dr. Mackintosh was the author of an elementary work on physic, and of various papers in the medical journals. He devoted a great portion of time and labour to the investigation of cholera, and has made some important contributions to its morbid anatomy, the results of which were communicated to the late scientific meeting at Liverpool. He was a zealous and active man, perhaps a little too *energetic* as a controversialist. He had a considerable class, and his death will be severely felt in the school with which he was connected, leaving, as we believe it does, a blank both in medicine and midwifery."

Dr. M. was chiefly known in the United States, by his systematic work on the "Principles of Pathology and Practice of Physic," edited by Dr. Morton, of Philadelphia, and used as a text book by the students of the Cincinnati College.

*M. Alibert.*—We avail ourselves of the following notice, found under the editorial head of the Philadelphia Medical Examiner; which we presume sets forth correctly, the leading facts of the life and character of this distinguisned French physician. He died in Paris, in November, at the age of 70.

"He was born in the village of Ville-franche, in what was formerly Haute Guyenne, in France. He was early destined for the Church, but the troubles of the revolution breaking out about that time, directed his attention in another channel. He removed to Paris, and became a pupil in the *Normal* school, an institution which, during its brief existence, gave considerable impulse to national education. It was not until this school was closed, that Alibert devoted his exclusive attention to medicine. The peculiar qualities of his mind, and the singular benevolence of his disposition, peculiarly adapted him for the exercise of the active duties of his profession. Many of his productions, and particularly those by which he is best known, were begun and completed during the first few years of his practice. We may mention, among others, his Treatise on Malignant Fever, which has been long regarded as a standard work; his Essay on Therapeutics, based on the physiology of Bichat; a system of nosology, which first displayed that peculiar talent for classification, afterwards so conspicuous in his elaborate production on the Diseases of the Skin; to these we may add numerous memoirs, eulogies, and other papers prepared for the Medical Society of Emulation, of which he was one of the most distinguished and active members. The first sketch of his great work on Cutaneous Affections appeared in 1806. It was not until 1832, that it was finally completed. The skill, fidelity and effect with which the illustrations of this noble work are executed are well known. This was done by Alibert at his own risk, and at an expense, it is said, of 300,000 francs. Willan had already acquired a reputation in this department, and as works of art, doubtless his plates possess much merit; but the superiority of vivid and natural delineation appears by general consent to be allotted to Alibert. The descriptions of the latter are also rendered more graphic and highly interesting by the vivacity of his style, and the richness of his imagery. The prominent fault of his nomenclature is excess of refinement, and of ingenuity, which, unfortunately, is better calculated to fatigue than to assist the memory of the student.

"His Treatise on the Passions, a work of a more purely literary character, was written at a later period. It is but justice to Alibert to confess, that in treating of the Passions, he has adopted a higher moral tone than is characteristic of the French School in general, and that his views appear to be much influenced by his early religious impressions.

"For the last twenty years of his life, Alibert occupied the post of physician-in-chief to the Hospital of St. Louis. This was the great scene of his labors and of his glory. Here he established his clinic on Cutaneous Diseases, and here in the open air, like one of the ancient academic sages, drawing his pupils around him, he exhibited and explained to them the cases, the number of which sufficed to illustrate the most exact classification, and by the power of his eloquence recreated this, his favorite branch of scientific investigation.

"In his private character, Alibert was distinguished for benevolence and kindness. His especial delight was giving aid and countenance to the junior members of the profession, and he regarded his pupils as his children. In his professional relations he was liberal and disinterested; all who sought his aid, whether rich or poor, were received with courtesy and kindness; and the importance of the malady, not the rank of his patient, was the rule and measure of the attention which he paid the case. Some of his more direct acts of benevolence were managed with extreme delicacy. If he discovered any artist, or literary man, who was reduced in his circumstances, he sent by some unknown hand, and under a fictitious name, considerable sums of money. Where the transaction was open, and the pride of the recipient made him insist on giving his note, it was either destroyed or cancelled. More than one hundred obligations thus mutilated were found among his papers.

"*Eloges* were delivered over his grave by MM. Pariset and Cruveilhier."

*Dr. Ives.*—From the journal just quoted, we extract the following annunciation of the death of this respectable physician:

"We announce with regret the death of Dr. Ansell W. Ives, of New York, after a protracted illness of intense suffering. He expired on Tuesday evening, the 6th of February, of Neuralgia, under which distressing affection he had been laboring for the past year. For the last nine months he endured the most excruciating and unspeakable agony, under which he eventually succumbed.

Dr. Ives was favorably known to the medical public as the Editor of the American edition of Paris' Pharmacologia, to which work he made a number of valuable additions."

*Dr. Eberle.*—This gentleman, after a protracted illness, expired, on the 2d of February last, at his new residence in Lexington, having, a few months before, left his professorship in the Medical College of Ohio, for the corresponding chair in Transylvania University. As a lecturer Prof. E. was respectable, but his fame rests more on his writings than his prelectiones. These are too well known and extensively disseminated throughout the United States, to require an enumeration. Their characteristics are good sense, fulness of detail, and the absence of *ultraism.* Dr. Eberle's habits were those of laborious bibliothecal research, and patient acquisition. The German was his mother tongue, and he cultivated the study of the medical literature of that Empire, more, perhaps, than any other American physician. His Materia Medica and Practice of Physic are works of decided and general utility. His age, at the time of his death, was 49. Of the nature of his disease, we are not correctly informed, but have understood it was apoplexy. D.

